# Early-stage endometrioid carcinoma with MSH6 protein deficiency: pitfalls in the diagnostic interpretation of microsatellite instability

**DOI:** 10.3389/fonc.2025.1520500

**Published:** 2025-05-21

**Authors:** Cheng Wang, Dongni Liang, Wei Wang, Wei Kuang, Juan Zou, Jing Zeng, Min Feng

**Affiliations:** ^1^ Department of Pathology, West China Second University Hospital, Sichuan University, Chengdu, Sichuan, China; ^2^ Key Laboratory of Birth Defects and Related Diseases of Women and Children, Ministry of Education, Chengdu, Sichuan, China; ^3^ Department of Gynecology and Obstetrics, West China Second University Hospital, Sichuan University, Chengdu, Sichuan, China

**Keywords:** Lynch syndrome, mismatch repair, endometrial carcinoma, microsatellite instability, minimal microsatellite shift

## Abstract

**Objective:**

Microsatellite instability (MSI)/mismatch repair (MMR) protein testing is important for Lynch syndrome (LS) identification, prognostic stratification, and immune checkpoint inhibitor screening in many solid malignancies. MSH6, an MMR protein, is less studied in LS, and the exact mechanism of inconsistent MSI and MMR results among endometrial cancer (EC) patients who are carriers of MSH6 mutations remains unclear. The aim of this study was to identify the molecular patterns and clinicopathological characteristics of MSH6 protein-deficient LS-related EC and to further investigate possible causes of discordant MSI and IHC results in *MSH6* variant carriers.

**Methods:**

Twenty-seven patients who were diagnosed with EC with only MSH6 protein deficiency from 2021 to 2023 at West China Second University Hospital were enrolled. PCR capillary electrophoresis (PCR-CE) was performed in all cases and further next-generation sequencing (NGS) was performed in non-MSI-high cases. Data on immunohistochemistry (IHC) markers, microsatellite shift patterns, and molecular profiles were further reviewed by an experienced molecular pathologist.

**Results:**

Among the 27 patients, 14 (51.9%) cases were found to be non-MSI-high, while only 8 of 14 (57%) cases successfully underwent NGS and ultimately incorporated into our study. All patients who were MSH6 protein negative were diagnosed with early-stage endometrioid carcinoma (EC), with a median age of 55 years (range 48–67 years). We reanalyzed the shift of all microsatellite loci and found one case with an additional unstable locus. Minimal microsatellite shifts (one to three nucleotide shift) were observed in all cases (100%), which occurred in mononucleotide markers from BAT 25 or BAT 26. Nevertheless, 3 of the 8 patients (37.5%) displayed MSI-H by NGS, which revealed truncating mutations in the MSH6 gene in exon 4 in 62.5% (5/8) of the patients, including nonsense mutations (37.5%), frameshift insertions (12.5%), and frameshift deletions (12.5%). The proportion of cases correctly classified (as determined via IHC markers) by MMR genomic status was greater (100%) than that correctly classified by PCR-CE (12.5%) in cases of MSH6 truncating variation. In addition, NGS (37.5%) had a higher MSI-H detection rate than PCR-CE (12.5%) in evaluating MSI status.

**Conclusion:**

Carriers of a germline pathogenic MSH6 variant are more likely to develop EC at an advanced age, and a non-MSI-H phenotype with minimal microsatellite shift is frequently observed only when the MSH6 protein is lost. This atypical MSI pattern is often overlooked, potentially increasing the risk of underdiagnosis of LS.

## Introduction

Lynch syndrome (LS) is an autosomal dominant inherited disorder characterized by an increased risk of developing colorectal cancer (CRC) in affected families, as well as extracolonic tumors in the endometrium, ovary, stomach, small intestine, urothelium, hepatobiliary system, and other organs (1, 2). Endometrial cancer (EC) is the most common extraintestinal tumor in women with LS, with a lifetime risk of approximately 40-60% (3, 4). The pathogenesis of LS is related to pathogenic germline variations in DNA mismatch repair (MMR) genes (*MLH1, MSH2, PMS2 and MSH6*) *(* 5). The MMR system can involve multiple mismatch repair proteins in DNA repair, including the MutS (MSH2, MSH3, MSH6, etc.) and MutL (MLH1, MLH3, PMS1, and PMS2) families. Among them, MLH1, MSH2, MSH6, and PMS2 are the dominant proteins of MMR. Mutations in DNA repair genes result in the accumulation of errors in microsatellite sequences so that they become either longer or shorter. The insertion or deletion of repetitive units and deficient mismatch repair (dMMR) during the DNA replication process can cause changes in the length of microsatellite alleles; this molecular phenotype is called microsatellite instability (MSI). Therefore, MSI and MMR are considered hallmarks of LS-related CRC and EC.

The vast majority of LS-associated tumors presents MSI due to their DNA MMR deficiency. A large number of studies have shown that tumors with dMMR/MSI molecular characteristics exhibit increased tumor antigen load due to high-frequency gene mutations, inducing infiltration of killing T lymphocytes and high expression of corresponding immunosuppressive molecules, resulting in a good response to corresponding immunotherapy (6). Therefore, dMMR/MSI detection for EC molecular classification, Lynch syndrome screening and immunotherapy prediction are crucial. Currently, universal screening for Lynch syndrome of all newly diagnosed endometrial cancer patients has been advocated in several clinical recommendations, including The Society of Gynecologic Oncology (SGO) and American College of Obstetricians and Gynecologists (ACOG). Specifically, they recommend the process of molecular evaluation of patients at risk for Lynch syndrome as follows: molecular tumor screening with immunohistochemistry (IHC) for MMR genes expression and/or microsatellite instability followed by germline genetic testing if the screening test is positive (7).

Although immunohistochemistry (IHC) and PCR capillary electrophoresis (PCR-CE) have a high concordance of results in CRC, several studies have shown that the PCR assay used for LS screening has a high false negativity rate and, particularly, a low detection sensitivity for the loss of MSH6 protein (8, 9). This may be related to the function of the MSH6 protein, which is involved in the repair of both single-base mismatches and insertion/deletion loops but is not absolutely required for MMR activity (10). In the absence of MSH6, the MSH3 protein can partially replace the MSH6 repair function and protect against DNA accumulation (11). On the other hand, up to 80% of dMMR tumors are attributable to somatic events and are, therefore, unrelated to LS (12, 13). Germline mutations in the *MSH6* gene account for approximately 15-30% of cases of hereditary nonpolyposis colorectal cancer (HNPCC), whereas *MSH6* germline mutations are seemed to be more common in EC than in CRC in some existing studies (14). Goodfellow et al. also showed that mutations in mismatch repair genes, especially MSH6, are closely related to the occurrence of EC (15). However, *MSH6* is less studied in molecular LS screening, the frequency of mutations in this gene may be largely underestimated, and the frequency and exact mechanism of inconsistent MSI and MMR results among carriers of *MSH6* mutations are still unclear. LS screening in EC patients is crucial for identifying which patients should be offered genetic counseling and genetic testing to prevent further LS-related cancers. Given the phenomenon of inconsistency of IHC and PCR-CE results, especially the relatively low sensitivity of MSI testing in EC patients with MSH6 germline mutations, which means tumors with MSH6 variants are particularly prone to discordant MMR/MSI, LS families with *MSH6* mutations may be underdiagnosed using traditional diagnostic criteria.

Therefore, in this study, we conducted an in-depth analysis of eight LS-related EC patients who were MSH6 protein negative and had germline *MSH6* truncating mutations, and described the molecular and clinical findings, aiming to explore and analyze the inconsistency and reasons for the loss of MSH6 protein and MSI PCR-CE, as well as the patterns of microsatellite shifting in these patients.

## Materials and methods

### Study population

**Figure 1:**
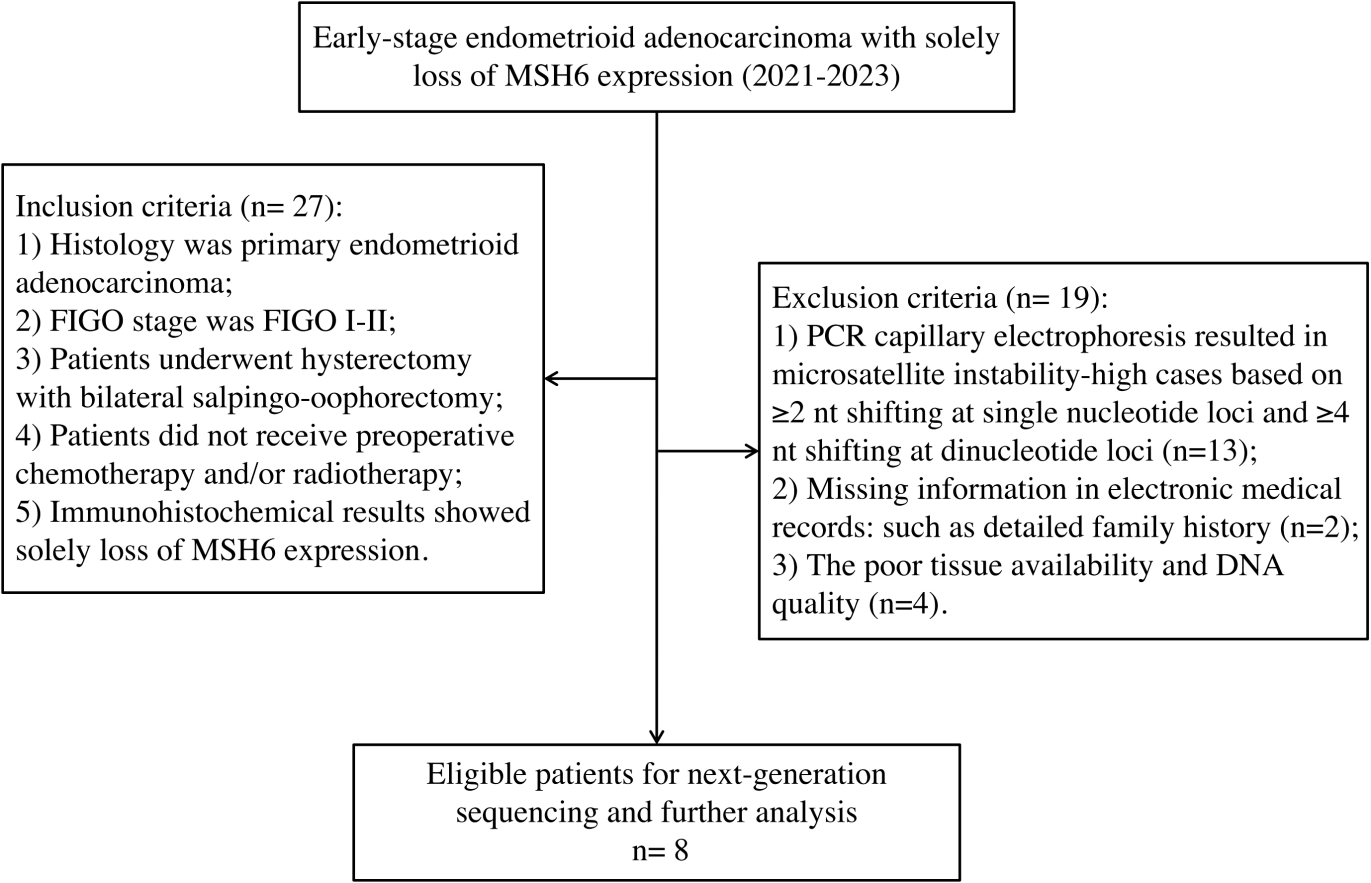
The study diagram of the selection process.

Patients with early-stage endometrioid carcinoma diagnosed with solely loss of MSH6 protein between January 2021 to December 2023 at West China Second University Hospital of Sichuan University were selected (n=27). All patients underwent hysterectomy with bilateral salpingo-oophorectomy and did not receive any preoperative chemotherapy and/or radiotherapy. Cases with non-MSI-H results by PCR-CE analysis were selected to further undergo NGS. Finally, only 8 cases successfully underwent NGS and ultimately incorporated into our study. The remaining non-MSI-H samples were failed to undergo NGS, mainly due to the lack of a detailed family medical histology, a low tumor tissue component that did not reach sufficient tumor concentration even after tumor cell enrichment, or poor DNA quality. According to the Amsterdam II criteria and Bethesda guidelines, electronic medical records, and detailed family history, oncologists made the clinical diagnosis of LS. The inclusion and exclusion criteria of our study were shown in **Figure 1**. The study was performed under a protocol approved by the institutional review board of the Ethics Committee of West China Second University Hospital of Sichuan University (protocol 2023037). Written informed consent was obtained from all the participants prior to the publication of this study.

### IHC staining

Representative formalin-fixed, paraffin-embedded (FFPE) tumor tissue blocks were used to make 4-μm sections. The EnVision system was used for visualization as previously described (16). The IHC staining antibodies used included MLH1, PMS2 (1:100; clone EP51), MSH2 (1:1000; clone MX061), MSH6 (1:1400; clone MX056), ER (ready-to-use, clone SP1), PR (ready-to-use, clone SP2), HER-2 (ready-to-use, clone 4B5), and p53 (1:600, clone MX008) antibodies. The results of the expression of MMR proteins were further interpreted as ‘retained’ and ‘lost’ by determining the intensity of staining in tumor cells relative to internal controls (lymphocytes, mesothelial cells, and glandular cells). Tumor tissues with surrounding normal tissues showed nuclear staining, which was considered retained staining. Complete loss of staining was defined as tumor cell nuclei without staining and normal nuclei of surrounding tissues with staining.

### Analysis of MSI by PCR-CE

MSI status was assessed by comparing tumor and matched normal DNA samples via PCR-CE analysis. In brief, the NCI-recommended panel was used for MSI analysis and contained two mononucleotide loci (BAT25 and BAT26) and three dinucleotide loci (D2S123, D5S346, and D17S250). All slices of FFPE tumor tissue specimens were used for testing after confirmation and enrichment of adequate tumor cells (>30%). Microsatellite instability high (MSI-H) was defined as the presence of two or more instability loci; microsatellite instability low (MSI-L) was defined as the presence of only one locus; and MSS was defined as the absence of instability at five loci. Two molecular pathologists reanalyzed the shift of all microsatellite loci. As previously study described, minimal microsatellite shift was further defined as a 1–3 nucleotide or base pair shift in the tumor DNA relative to the matched normal tissue at an involved locus, and a major shift was more than 3 microsatellite repeat shifts (14).

### Massively parallel sequencing

Paired germline and somatic tumor sequencing were performed on tumor and matched normal samples via 1021-gene panel targeted sequencing (**Supplementary Table**). In brief, somatic single nucleotide variants (SNVs) and insertions and deletions (InDels) were detected via MuTect (v1.14) and GATK (the Genome Analysis Toolkit, v3.4-46-gbc02625), respectively (17). Copy number alterations (CNVs) in the tumor were identified with CONTRA (Copy Number Targeted Resequencing Analysis, v2.0.8). The MSIsensor algorithm for the detection of somatic microsatellite changes computes the length distributions of microsatellites per site in paired tumor-normal sequence data, subsequently yielding a quantitative MSIsensor score; MSIsensor scores ≥10 presented with MSI-H, 3–9 presented with MSI-L, <3 presented with MSS (18).The tumor mutational burden (TMB) was defined as the number of somatic nonsynonymous SNVs and InDels per megabase (mut/Mb), TMB-H defined as ≥10 muts/Mb, <10 muts/Mb presented with TMB-L. The identified genetic variants were manually assessed via IGV (v2.13.1), and interpreted via current standards for variant classification according to American College of Medical Genetics and Genomics (ACMG) guidelines, and all germline mutations in patients in our cohort were classified as likely pathogenic or pathogenic according to ACMG criteria.

## Results

### Clinicopathologic characteristics

A total of 8 of 27 patients with solely loss of MSH6 protein successfully completed further massively parallel sequencing and had confirmatory germline testing to screen for LS. Among the 8 patients, pathogenic germline variants were identified in *MSH6* (100%), 4 (50%) harbored a frameshift mutation and 4 (50%) harbored a nonsense mutation. Seven (87.5%) patients had EC as their sole malignancy, and 1(12.5%) patient with a history of CRC was diagnosed with LS. Six patients (75%) had a family history of LS-related neoplasia (**Table 1**). All patients had been diagnosed with early stage endometrioid carcinoma (FIGO I-II), 6 (75%) were histologic grade 1, and 2 (25%) were grade 2 or 3. Additionally, 2 (25%) cases occurred in the uterine horn, and 6 (75%) in the uterine fundus. The median age of our cohort was 55 years (range 48–67 years), and the median tumor size was 2.5 cm (range 1.0-6.2) (**Table 2**).

**Table 1:** Summary of germline variations and cancer history in our cohort.

Case	Age	Germline variation	Type of variation	Personal history	Family history
050	57	MSH6, c.1804_1805delTC, p.S602Kfs*4	Frameshift Deletion	None	YB, LC
099	58	MSH6, c.3142C>T, p.Q1048*	Nonsense Mutation	None	None
153	56	MSH6, c.3514dupA, p.R1172Kfs*5	Frameshift Insertion	None	YB, CRC
015	48	MSH6, c.651dupT, p.K218*fs*1	Frameshift Insertion	CRC/BC	MU, CRC; ES, EC; PU, LIC
002	67	MSH6, c.3261dup, p.F1088Lfs*5	Frameshift Insertion	None	M, EC
587	53	MSH6, c.373A>T, p.K125*	Nonsense Mutation	None	None
736	55	MSH6, c.718C>T, p.R240*	Nonsense Mutation	None	M, LUC
892	48	MSH6, c.862G>T, p.E288*	Nonsense Mutation	None	ES, EC

M, mother; YB, younger brother; ES, elder sister; MU, maternal uncle; PU, paternal uncle; LIC, liver cancer; CRC, colorectal cancer; EC, endometrial cancer; LUC, lung cancer; BC, breast carcinoma; *, stop codon; fs, frame shift..

**Table 2:** Clinicopathological characteristics of endometrial cancer in our cohort.

Case	FIGO stage	Grade	Histology	Primary site	Tumor size (cm)	LVI	Myometrial invasion	IHC staining	CHO	RAD
050	I	G1	UEMC	UF	1.0	No	<50%	MSH6-, ER/PR++, p53wt	None	None
099	I	G3	UEMC	UF	6.2	Diffuse	≥50%	MSH6-, ER/PR+, p53wt	None	Yes
153	II	G3	UEMC	UF	3.0	Focal	<50%	MSH6-, ER/PR partial+, p53wt	Yes	Yes
015	I	G1	UEMC	UF	3.0	No	<50%	MSH6-, ER++, PR partial+, p53wt	None	None
002	I	G1	UEMC	UH	1.2	No	<50%	MSH6-, ER/PR+, p53wt, Her-2 (-)	None	None
587	I	G1	UEMC	UH	1.1	No	<50%	MSH6-, ER/PR+, p53wt, Her-2 (1+)	None	None
736	I	G1	UEMC	UF	2.8	Diffuse	<50%	MSH6-, ER/PR+, p53wt, Her-2 (1+)	Yes	None
892	I	G1	UEMC	UF	2.0	Focal	<50%	MSH6-, ER/PR+, p53wt, Her-2 (-)	None	None

UEMC, Uterine Endometrioid Carcinoma; LVI, Lymphatic vascular invasion; LYN, Lymphadenectomy; UF, Uterine fundus; UH, Uterine horn; IHC, Immunohistochemistry; CHO, Chemotherapy; RAD, Radiotherapy.

### Immunohistochemistry results

**Figure 2:**
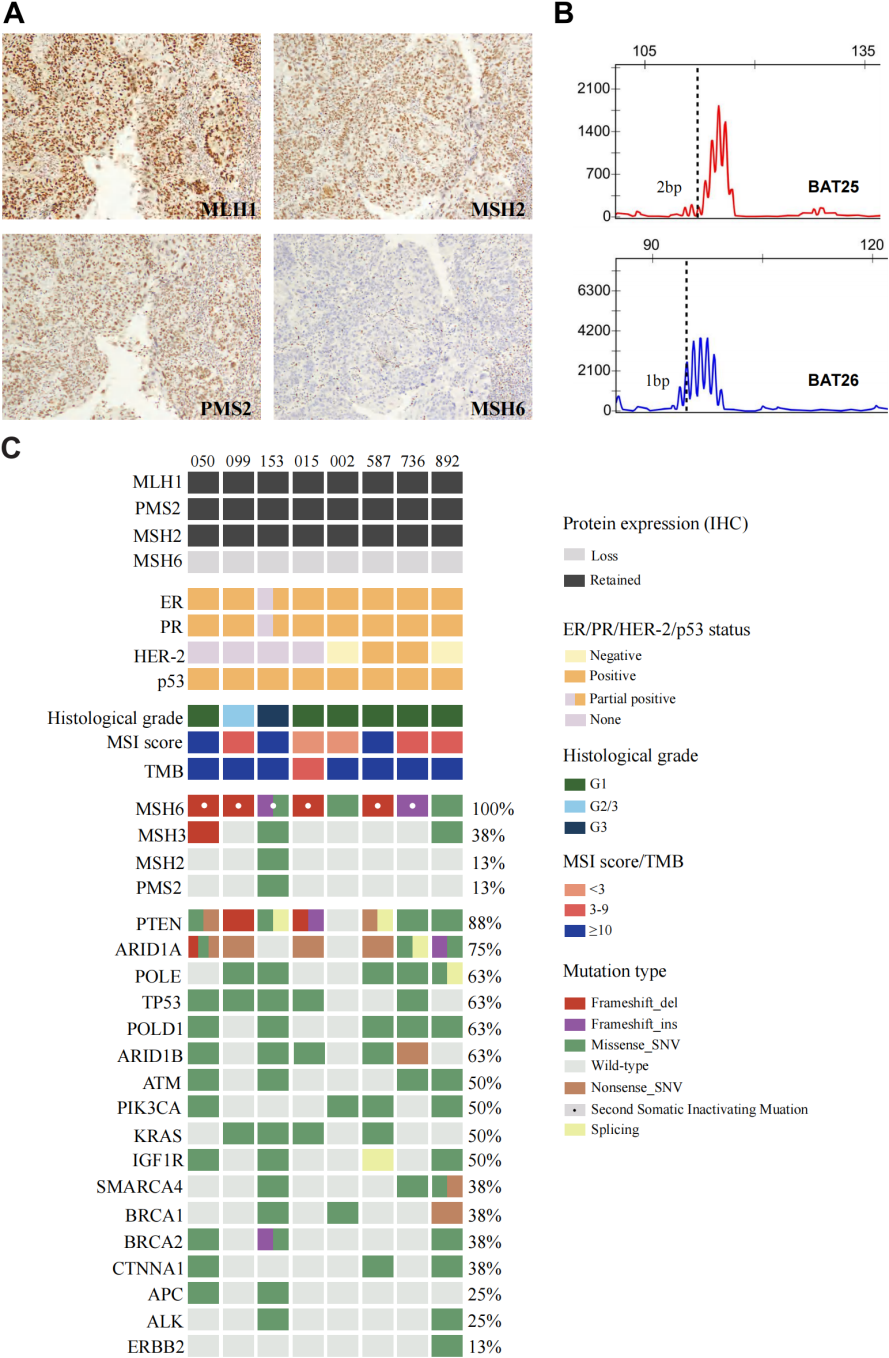
Positive staining results of **(A)** MLH1/PMS2/MSH2 and absence of MSH6 protein in endometrioid carcinoma of Case 153. **(B)** Microsatellite instability testing by PCR capillary electrophoresis (2B/3D panel). **(C)** Clinicopathologic and genomic characterization of endometrioid carcinoma in patients with Lynch syndrome related germline pathogenic variants.

Among the 8 tumors, 7 (87.5%) were ER/PR/p53-positive, and the remaining tumor (12.5%) was ER/PR partially positive and p53 positive. Four tumors had available HER-2 test results, 2 (50%) were positive, and the remaining (50%) were negative. An isolated protein loss of MSH6 was present in all patients (100%), with truncating mutations impairing protein function. Patient 153, harboring an *MSH6* c.5341dupA frameshift insertion, demonstrated complete loss of MSH6 (**Figure 2A**) and partial ER/PR positive (**Figure 2C**).

### Microsatellite instability analysis

PCR-CE analysis of 8 EC patients revealed a change of any length due to microsatellite repeat unit insertions or deletions in tumor tissue compared with normal tissue. The most representative MSI pattern was non-MSI. Specifically, 7 of the 8 (87.5%) patients presented with MSI-L status, which was inconsistent with the IHC results. A microsatellite repeated unit change (ranging from 1bp to 3bp) at mononucleotide markers from BAT25 or BAT26 was observed in these patients (**Table 3**). Minimal microsatellite shifts were found in all cases (100%) of microsatellite repeat unit changes that occurred in mononucleotides, including the only MSI-H case (case 153, **Figure 2B**), which slightly shifted at BAT25 and BAT26. Five of the 7 MSI-L cases (71.4%) with minimal microsatellite shifts occurred at the BAT25 locus of the single nucleotide marker and 2 (28.6%) occurred at BAT26.

**Table 3:** Summary of MMR gene mutations, MSI status (based on PCR), tumor mutational burden and MSI score (based on NGS) in our cohort.

Case	MMR somatic variations	Second hit in MMR genes	MSI status (instable loci)	MSIsensor score	TMB score (Muts/Mb)
050	MSH6, c.3261delC, p.F1088Sfs*2, AF: 22.6%; MSH6, c.3430dupA, p.M1144Nfs*20, AF: 7.5%	Yes	MSI-L (1bp, BAT25)	MSI-H (12.85)	TMB-H (99.84)
099	MSH6, c.928_929del, p.L310*, AF:9.1%	Yes	MSI-L (3bp, BAT25)	MSI-L (6.51)	TMB-H (71.04)
153	MSH6, c.3261_3262insC, p.F1088Lfs*5, AF:10.9%; MSH6, c.206C>A, p.A69E, AF:14.9%; MSH6, c.1688C>T, p.T563I, AF:12.8%; PMS2, c.1451C>A, p.P484H, AF:15.4%; MSH2, c.1597C>A, p.L533I, AF:13.9%	Yes	MSI-H^†^ (2bp, BAT26; 1bp, BAT25)	MSI-H (18.22)	TMB-H (365.76)
015	MSH6, c.3261delC, p.F1088Sfs*2, AF: 5.3%	Yes	MSI-L (1bp, BAT26)	MSS (1.21)	TMB-L (5.76)
002	MSH6, c.2419G>A, p.E807K, AF:7.9%	No	MSI-L (2bp, BAT25)	MSS (2.7)	TMB-H (46.08)
587	MSH6, c.2845delC, p.Q949Rfs*7, AF:36.2%	Yes	MSI-L (2bp, BAT26)	MSI-H (25.67)	TMB-H (90.24)
736	MSH6, c.3261_3262insC, p.F1088Lfs*5, AF:16.3%	Yes	MSI-L (2bp, BAT25)	MSI-L (9.28)	TMB-H (93.12)
892	MSH6, c.77G>A, p.R26K, AF:1.4%	No	MSI-L (1bp, BAT25)	MSI-L (3.42)	TMB-H (236.16)

MMR, Mismatch repair; MSI, Microsatellite instability; MSI-L, Microsatellite instability-low; MSI-H, Microsatellite instability-high; MSS, Microsatellite instability stable; MSIsensor scores ≥10 presented with MSI-H, 3–9 presented with MSI-L, <3 presented with MSS; TMB, Tumor mutational burden; TMB-H defined as ≥10 muts/Mb, <10 presented with TMB-L; *, stop codon; fs, frame shift. ^†^This was the result of the reanalysis (incorporating minimal shift into the criteria for instable locus interpretation).

### Genomic features

**Figure 3:**
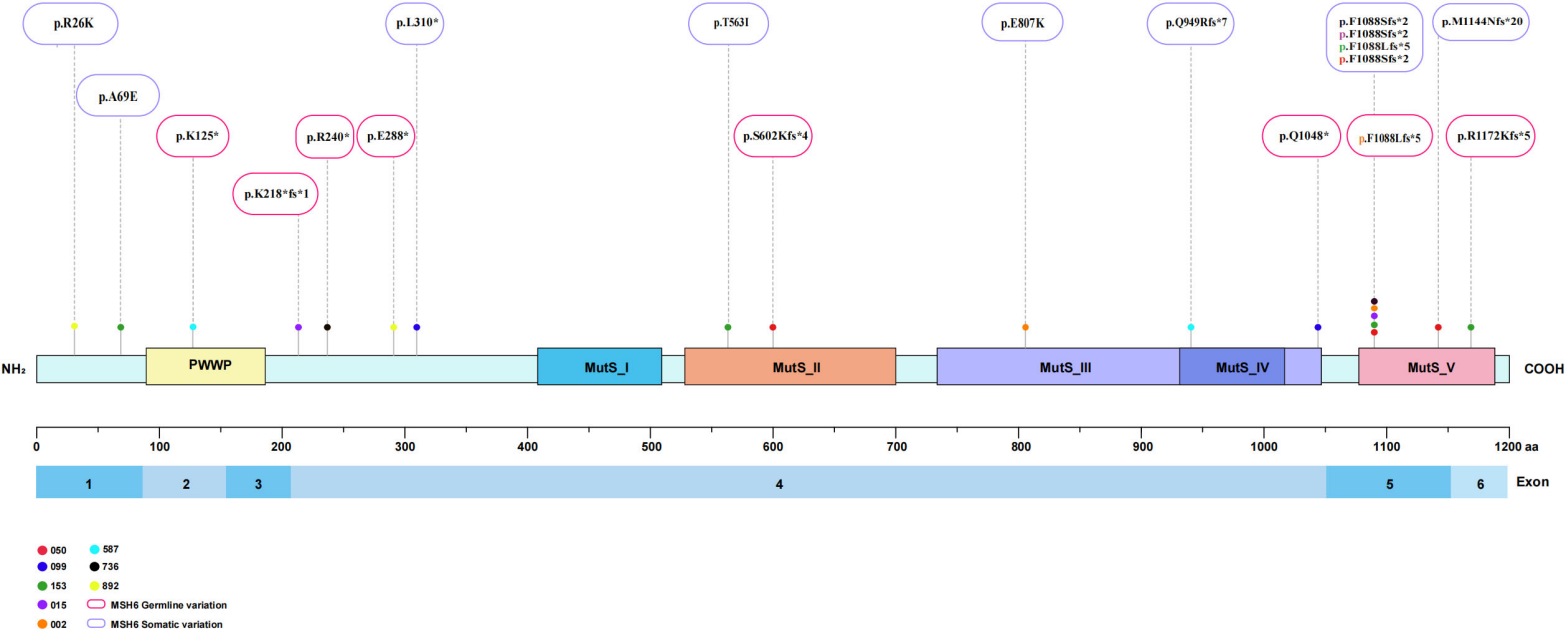
Structure of the MSH6 protein showing function and variants located in the study cohort. *MSH6* germline variations and somatic variations are marked red boxes and purple boxes.

Alterations in a repertoire of somatic mutations were confirmed via targeted sequencing of 1021 genes, including *PTEN* (88%), *ARID1A* (75%), *POLE/TP53/POLD1/ARID1B* (63%), and *ATM/PI3KCA/KRAS/IGF1R* (50%) (**Figure 2C**). All the EC patients whose MSH6 protein was abnormal according to IHC showed a second hit in the proposed LS-related gene. Four patients (cases 015, 050, 099 and 587) harbored frameshift deletions (p.F1088Sfs*, p.L310*, and p.Q949Rfs*), resulting in second somatic inactivation of *MSH6*, and 2 patients (cases 153 and 736) harbored frameshift insertion (p.F1088Lfs*). Recurrent passenger mutation in an *MSH6* exon 5 coding microsatellite, *MSH6* F1088fs*, was observed in 50% (4/8) of all patients (**Table 3**, **Figure 3**).

**Figure 4:**
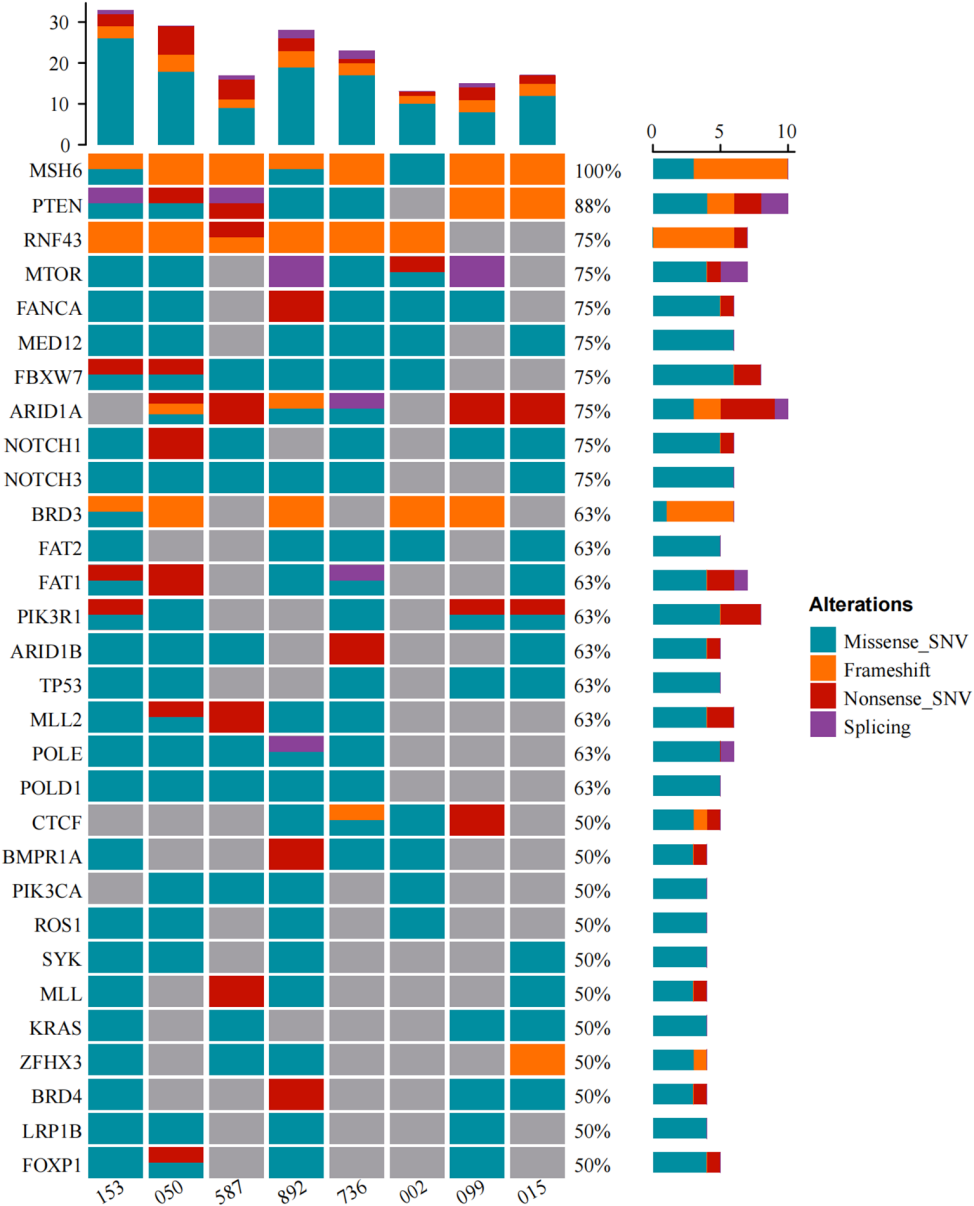
Somatic mutation landscape of our study cohort.

MSI status was further analyzed via next-generation sequencing (NGS), and 3 of 8 cases (37.5%) displayed MSI-H (**Figure 2C**), whereas cases 050 and case 587 were defined as MSI-L when one unstable mononucleotide marker was detected via PCR-CE. Patient 099 presented evidence of MSI-L by PCR analysis but had a low MSIsensor score of 6.51, which was deemed MSS. The sole case (case 015, **Table 3**) considered to be MSI-L/MSS with TMB-L was a 48-year-old woman with a personal history of colorectal and breast cancer and family members who had a history of LS-related cancer. The tumor was a well-differentiated EC occurring at the uterine fundus with a low tumor mutational burden (TMB score=5.76 Muts/Mb) and MSS (MSIsensor score=1.21). In our cohort, the TMB was > 10 in all patients except patient 015, where patients 153 and 892 had a high TMB or ultramutated phenotypes (TMB score>100 Muts/Mb). A minority (25%, 2/8) of the tumors had multiple *MSH6* mutation types, many tumors had only one mutation, and the molecular landscape of all the patients is presented in **Figure 4**. Taking IHC as the reference, the proportion of cases correctly classified by MMR genomic status was greater (100%) than classified by PCR-CE (12.5%) in cases of *MSH6* truncating variation. In addition, NGS (37.5%) testing had a higher MSI-H detection rate than PCR-CE (12.5%) in the evaluation of MSI status.

## Discussion

In the current study, we performed NGS of 8 out of 27 LS-EC patients with only MSH6 protein negative from the largest gynecological oncology center in Southwest China. Of the 8 LS-EC patients, 7 had MSI-L by PCR-CE and 5 had MSI-L/MSS by NGS. The rate of discordance between IHC-MMR and PCR-MSI in ECs was 87.5%, which was significantly higher than that of the NGS-MSI strategy (62.5%). *MSH6* mutations were demonstrated by next-generation sequencing (NGS) in 8 of 27 patients with loss of MSH6 protein expression, and all the mutations were truncating mutations, which are generally classified as pathogenic. The evidence from EC patients with LS in our cohort indicated that pathogenic germline mutations in *MSH6* are more frequently present as non-MSI-H status (including MSI-L and/or MSS). This may reveal a pitfall for LS individuals with MSI identified by either PCR or multigene panel testing in ECs with *MSH6* germline truncating mutations.

*MSH6*, an MMR gene also known as G/T mismatch binding protein, is located at 2p16, a site not far from *MSH2*, with a total DNA length of 23,806 bp and 10 exons (19). Compared with those in CRC, *MSH6* germline mutations are more common in EC, as demonstrated in many studies (20). Previous studies have shown that the incidence of EC is 26-fold greater in women who carry *MSH6* pathogenic variants than in the general population (21). In a study including 22 EC patients with LS, who typically harbor MMR genetic germline mutations, 55% had *MSH6* and *PMS2* mutations, which is higher than previously reported (22). However, given the scarcity of reports of mutations in the *MSH6* gene in molecular LS screenings, mutations in this gene could be largely underestimated. More importantly, more evidences has indicated that the risk for EC is significantly greater in women with *MSH6* pathogenic variants than in those with *MLH1* or *MSH2* variants, and the cumulative risk for the diagnosis of EC throughout their lifetime is 16%-49% (23, 24). Therefore, determining *MSH6* variant pathogenicity is of significant clinical importance, particularly for predicting cancer risk. According to the available data, the most frequent *MSH6* mutation occurs in exon 4, and the remaining exons have a lower mutation frequency (25). Truncating mutations in *MSH6* have been identified in patients with hereditary LS and are generally classified as pathogenic. Truncating mutations can introduce a premature stop codon that results in a C-terminal truncated form of the protein, including partial or complete deletion of the highly conserved *MutS* structural domain. Total or partial loss of this structural domain results in loss of ATPase activity, which impairs DNA binding and mismatch repair functions (26, 27). In our study, massively parallel sequencing analysis revealed truncating mutations in the *MSH6* gene in exon 4 in 62.5% (5/8) of patients, including nonsense mutations (37.5%), frameshift insertions (12.5%) and frameshift deletions (12.5%). What draws more of our attention is that these cases presented a non-MSI-H pattern on PCR-CE analysis, or displayed a relatively lower degree of MSI, which is consistent with the recent research results in CRC by Helderman et al (28).

Previous studies in yeast have shown that mutations in *MSH6* do not lead to dinucleotide repeat instability but instead lead to weak single nucleotide repeat instability and significantly increase the accumulation rate of base substitution mutations (29). In addition, studies of *MSH6* mutant mice have shown that these mice have significantly increased cancer susceptibility and that the tumors in these mice do not exhibit repeat instability, similar to the results observed in yeast (30). Researchers have reported that mice harboring *MSH6* mutations not only have a reduced life expectancy, but also develop a variety of unrelated tumors involving multiple organ systems. In the following years, several *in vitro* and *in vivo* studies established the association of HNPCC-related tumors with low MSI with germline mutations in *MSH6*, further confirming that mutations in *MSH6* alone were not sufficient to cause MSI in CRC cell lines (31, 32). These results suggest that *MSH6* mutations may contribute to cancer susceptibility, but the tumors produced may differ from those observed in kinases that inherit *MSH2* and *MLH1* mutations, at least in terms of their microsatellite instability phenotype. For eukaryotes, in the process of DNA mismatch repair, *MutS* homologous dimer (with mismatch binding activity) and *MutL* homologous dimer (capable of interacting with proteins) combine to form a tetramer complex and work synergistically. And there are a number of heterodimeric *MutS* homologs, the most important of which are *MutSα* (MSH2/MSH6) and *MutSβ* (MSH2/MSH3); the former recognizes one or two base unpaired sites, while the latter recognizes longer insertion-deletion loops with up to more than ten nucleotides (19). The MSH6 protein is involved in the repair of both single-base mismatches and insertion/deletion loops but is not absolutely required for the MMR system. In the absence of MSH6, the MSH3 protein can partially replace the MSH6 repair function, and the MSI profile is not present as MSI-H. Therefore, non-MSI-H (MSS/MSI-L) phenotype could not be considered as an exclusion criterion for MMR gene monitoring, especially for MSH6.

PCR-CE is the gold standard for assessing MSI, including 5 or 7 loci, whereas NGS-based methods can examine hundreds to thousands of target microsatellite loci, allowing for a more comprehensive assessment. Our data revealed that 7 (87.5%) LS individuals with solely MSH6 loss presented non-MSI-H disease by PCR, but 5 cases (62.5%) were identified by NGS. In our cohort, we identified only one patient with MSI-H status (using PCR-CE assay), and the BAT25 locus may not be a truly unstable locus if a 2bp changing is used as a criterion for unstable loci interpretation; thus, Patient 153 exhibited an MSI-L pattern. Our findings may provide direct evidence that a subset of primary ECs that develop in the context of an *MSH6* germline pathogenic mutation harbor features of non-MSI-H, including MSS or MSI-L, which is inconsistent with previous observations (16). An MSIsensor score of 10 reliably identifies the MSI-H status of solid tumors at various primary sites, and again, we used this criterion (33). TMB-H was found in all three cases with MSIsensor scores >10 (cases 050, 153 and 587), and *MSH3* variants were found in 2 of them, suggesting that MSIsensor performs well in MSH6-deficient ECs. Middha et al. reliably assessed pan-cancer microsatellite instability using MSK-IMPACT assay and found that MSIsensor may be sensitive for the MSH6-equivocal EC, suggesting that MSIsensor performs well in MSH6-deficienct tumors. Our massively parallel sequencing was also similar to MSHK-IMPACT, and therefore, we adopted MSIscore greater than or equal to 10 as a criterion for MSI-H interpretation (18). Compared with PCR methods, Simultaneous MSI detection by NGS not only saves resources efficiently, but also may be more sensitive to dMMR and may identify MSI-H in a wide range of cancers not typically screened.

Notably, among different solid tumors, the number of microsatellite instability nucleotide shifts varies by PCR-CE, including major microsatellite repeat shifts and minimal shifts (34). At present, although there is no unified guideline for the definition of minimal microsatellite shift, it was defined by shift of 1 to 3 microsatellite nucleotide repeats at an involved locus in most studies. Moreover, MSI-H ECs have a significantly greater frequency (52%) of minimal microsatellite shift (14, 35). A comparing the MSI status of EC and CRC revealed that 53% of MSI-H-type EC cases presented an average of 1–2 small nucleotide changes, whereas approximately 80% of MSI-H-type CRC cases presented an average of 6 nucleotide changes (36). Therefore, minimal microsatellite shifts are more likely to occur in EC. The subtle changes caused by minimal microsatellite shifts are easily overlooked when interpreting PCR-CE results, leading to false-negative results. Therefore, minimal microsatellite shifts are also considered one of the main reasons for the high inconsistency rates of IHC and PCR-CE in EC. According to the criteria of minimal shifting, minimal microsatellite shifts were found in all cases (100%) of tumors with only MSH6 loss, and instability loci occurred only at single nucleotide sites in our study. Our results are higher than those of previous studies (30%) (14, 37), suggesting that ECs with the loss of MSH6 alone had a greater chance of minimal shift. Therefore, the identification of minimal microsatellite shifts is crucial for accurate interpretation of microsatellite instability PCR data in EC in terms of clinical diagnosis.

The major limitation of MSI testing is that it is less accurate in identifying EC patients with the *MSH6* mutations. Minimal microsatellite shifts are may be another major reason; if the MSI status is evaluated on the basis of minimal microsatellite shifts, the detection sensitivity of PCR-CE and its consistency with IHC can be improved. Since the MSH6 protein is not involved in the repair of mismatches of dinucleotides in length, and consequently, the 2B/3D panel (NCI recommended) often shows an MSS in MSH6-deficient tumors, mononucleotide repeats are recognized as being more sensitive and specific for determining of the MSI status in these tumors (38). MSH6-deficient tumors are, therefore at risk of being misclassified as MSI-L or MSS, depending on the markers chosen. Some investigators have recommended assessing the MSI status of MSH6-deficient tumors via a panel of 5 mononucleotide markers, including NR21, BAT25, BAT26, NR24 *and* NR22 (Pentaplex Assay) (39). In addition, for ECs with superficial muscle layer infiltration, collecting a sufficient proportion of tumor cells during the detection process is difficult, which may also negatively affect the MSI results. Overall, researchers need to be aware that PCR-CE testing is challenging when used to detect *MSH6* truncated variant carriers.

We explored the clinicopathological characteristics of ECs in LS patients. All patients were diagnosed with endometrioid carcinoma at an early stage (100%), and the majority were well differentiated (75%). Notably, the median age of the 8 LS individuals with an isolated germline *MSH6* mutation in our cohort was 56.5 years (range from 48 to 67 years). A published study revealed that EC patients with *MSH6* gene mutations had a greater mean age of onset (58 years) than did patients with *MLH1* or *MSH2* gene mutations (49 years) (40). Despite the small number of cases in our study cohort, a delay in the age of onset of EC, which is characteristic of *MSH6* mutations, is also well supported. LS patients are usually clinically diagnosed according to the Amsterdam or Bethesda criteria. However, the current clinical criteria for patients with LS (who typically harbor *MSH2* and/or *MLH1* germline mutations) were deemed insensitive for identifying *MSH6* mutation carriers. Furthermore, owing to potential challenges in gathering family information and the absence of distinctive clinical phenotypes, the diagnostic rate may be much lower than the actual incidence. Thus, criteria for a diagnosis of *MSH6*-related LS were established that differed from the Amsterdam major and/or minor criteria and should incorporate the unusual phenotypes of patients with an isolated germline *MSH6* mutation, such as those patients who are older at the time of diagnosis of the primary malignancy (41). IHC is still the initial screening tool for the detection of the involved MMR protein. With respect to the retention of MSH2 and MLH1 expression and the lack of MSH6 alone, MSI analysis using a panel composed of mononucleotides alone (Pentaplex Assay) is recommended instead of the standard 2B/3D panel. In the case of non-MSI-H, germline mutation analysis of *MSH6* is needed, especially in the context of a positive family history. Even if germline mutation analysis of *MSH6* is negative, the proband and his family members will still require strict cancer surveillance.

A limitation of this study was the retrospective, highly selective nature of this cohort. Additionally, this study is based on a relatively small sample size from a single center, and a well-designed multicenter study is still needed to demonstrate the incidence of *MSH6* gene mutations and the clinical phenotype, which is one of our ongoing studies in China. The MSIsensor score cutoff value varies somewhat from cancer to cancer, and further large-sample studies are needed. Our study expands the spectrum of the known germline mutations of the *MSH6* gene in EC patients who are MSH6 protein negative. A multicenter trial with a larger number of EC patients with LS is needed to clarify the biological impact of these mutations on susceptibility to LS and their impact on the effectiveness of anti-PD-1 treatment.

In conclusion, this study highlights that the diagnosis of LS caused by pathogenic germline MSH6 variants may be complicated by inconsistent results in terms of the IHC and PCR-CE phenotypes. The molecular and clinical data of these patients add to our understanding of the clinical implications of MSH6 germline variants. We explored a variety of causes for discordant MSI and IHC results in MSH6 variant carriers. Compared with other methods, IHC is widely available and not expensive and may confer an advantage over PCR-CE due to the lower sensitivity of PCR-CE for MSH6-deficient tumors. In addition, MSI analysis using a panel composed of mononucleotides is recommended instead of the standard 2B/3D panel when tumors lack MSH6 alone, and germline mutation analysis of MSH6 is mandated.

## Data Availability

The datasets presented in this study can be found in online repositories. The names of the repository/repositories and accession number(s) can be found below: https://www.ncbi.nlm.nih.gov/, Sequence Read Archive (SRA) database (PRJNA990462).
