# Supplementary material for: Early-stage endometrioid carcinoma with MSH6 protein deficiency: pitfalls in the diagnostic interpretation of microsatellite instability
Source: Front Oncol. 2025 May 21;15:1520500. doi: 10.3389/fonc.2025.1520500 (PMC12133529; doi:10.3389/fonc.2025.1520500)
Supplement: Supplementary file 1 [file Table1.docx]

Supplementary Table S1. List of target regions of the pan-cancer 1021-gene panel.

| **170-gene coding sequence** | | | | | | | | | | | | | | | | |
| --- | --- | --- | --- | --- | --- | --- | --- | --- | --- | --- | --- | --- | --- | --- | --- | --- |
| *ABL1* | *BRD3* | | *CDKN2B* | | *FAT1* | | | *HDAC1* | | *MCL1* | *NOTCH3* | | *PTEN* | | *SYK* | |
| *ABL2* | *BRD4* | | *CHEK1* | | *FBXW7* | | | *HDAC4* | | *MDM2* | *NOTCH4* | | *PTPN11* | | *TMPRSS2* | |
| *AKT1* | *BTK* | | *CHEK2* | | *FCGR2A* | | | *HGF* | | *MDM4* | *NRAS* | | *RAF1* | | *TOP1* | |
| *AKT2* | *C11orf30* | | *CRKL* | | *FCGR2B* | | | *HRAS* | | *MED12* | *NTRK1* | | *RARA* | | *TP53* | |
| *AKT3* | *C1QA* | | *CSF1R* | | *FCGR3A* | | | *IDH1* | | *MET* | *NTRK3* | | *RB1* | | *TSC1* | |
| *ALK* | *C1S* | | *CTNNB1* | | *FGFR1* | | | *IDH2* | | *MITF* | *PALB2* | | *RET* | | *TSC2* | |
| *APC* | *CBL* | | *DDR1* | | *FGFR2* | | | *IGF1R* | | *MLH1* | *PDGFRA* | | *RHEB* | | *VEGFA* | |
| *AR* | *CCND1* | | *DDR2* | | *FGFR3* | | | *IL7R* | | *MLH3* | *PDGFRB* | | *RHOA* | | *VHL* | |
| *ARAF* | *CCND2* | | *DNMT3A* | | *FGFR4* | | | *INPP4B* | | *MPL* | *PDK1* | | *RICTOR* | | *XPO1* | |
| *ATM* | *CCND3* | | *EGFR* | | *FLCN* | | | *IRS2* | | *MS4A1* | *PIK3CA* | | *RNF43* | | *XRCC1* | |
| *ATR* | *CCNE1* | | *EPHA2* | | *FLT1* | | | *JAK1* | | *MSH2* | *PIK3CB* | | *ROCK1* | |  | |
| *AURKA* | *CD274* | | *EPHA3* | | *FLT3* | | | *JAK2* | | *MSH3* | *PIK3R1* | | *ROS1* | |  | |
| *AURKB* | *CDH1* | | *EPHA5* | | *FLT4* | | | *JAK3* | | *MSH6* | *PIK3R2* | | *RPS6KB1* | |  | |
| *AXL* | *CDK13* | | *ERBB2* | | *FOXA1* | | | *KDR* | | *MTOR* | *PMS1* | | *SMARCA4* | |  | |
| *BAP1* | *CDK4* | | *ERBB3* | | *FOXL2* | | | *KIT* | | *MYC* | *PMS2* | | *SMARCB1* | |  | |
| *BCL2* | *CDK6* | | *ERBB4* | | *GAB2* | | | *KRAS* | | *MYD88* | *PRKAA1* | | *SMO* | |  | |
| *BRAF* | *CDK8* | | *ERCC1* | | *GATA3* | | | *MAP2K1* | | *NF1* | *PSMB1* | | *SRC* | |  | |
| *BRCA1* | *CDKN1A* | | *ERG* | | *GNA11* | | | *MAP2K2* | | *NF2* | *PSMB5* | | *STAT1* | |  | |
| *BRCA2* | *CDKN1B* | | *ESR1* | | *GNAQ* | | | *MAPK1* | | *NOTCH1* | *PTCH1* | | *STAT3* | |  | |
| *BRD2* | *CDKN2A* | | *EZH2* | | *GNAS* | | | *MAPK3* | | *NOTCH2* | *PTCH2* | | *STK11* | |  | |
| **851-gene hot exons** | | | | | | | | | | | | | | | | |
| *ABCA10* | | *CAPRIN1* | | *DMXL1* | | *GLYR1* | *LMAN1L* | | *NXF5* | | | *RALBP1* | | *STAG2* | | *UNC13A* |
| *ABCA8* | | *CARS* | | *DMXL2* | | *GMDS* | *LMBR1L* | | *OBP2A* | | | *RAPGEF2* | | *STAT4* | | *UNC13D* |
| *ABCB7* | | *CARS2* | | *DNAH10* | | *GNPTAB* | *LPCAT4* | | *OBP2B* | | | *RARB* | | *STAT6* | | *UNC5D* |
| *ABCC8* | | *CASC4* | | *DNAH5* | | *GOLGA4* | *LPHN3* | | *OCA2* | | | *RASEF* | | *STK11IP* | | *USP12* |
| *ABCF2* | | *CASP8* | | *DNAH9* | | *GPAT2* | *LRBA* | | *ODZ3* | | | *RBM6* | | *STK31* | | *USP34* |
| *ACE* | | *CASP8AP2* | | *DNAJC11* | | *GPATCH2* | *LRP1B* | | *OR2T4* | | | *RBMX* | | *STX3* | | *USP39* |
| *ACER2* | | *CASQ2* | | *DNAJC9* | | *GPR114* | *LRP2* | | *OR4A15* | | | *RCC1* | | *SULT1A4* | | *USP45* |
| *ACOT11* | | *CATSPER2* | | *DNTTIP1* | | *GPR125* | *LRP4* | | *OR4C6* | | | *REC8* | | *SUPT5H* | | *USP48* |
| *ACPP* | | *CBFB* | | *DOCK11* | | *GPR133* | *LRRC16B* | | *OR5L2* | | | *REG1B* | | *SUPT6H* | | *VAV1* |
| *ACSL1* | | *CBX4* | | *DOCK3* | | *GPR144* | *LRRC2* | | *OR6F1* | | | *RELN* | | *SYCP2L* | | *VEZF1* |
| *ACSM5* | | *CCDC155* | | *DOT1L* | | *GPS2* | *LRRC7* | | *OSBPL10* | | | *RERE* | | *SYNE1* | | *VILL* |
| *ACSS3* | | *CCDC159* | | *DPP10* | | *GRIA3* | *LRRC72* | | *OTOA* | | | *RFWD2* | | *SYNE2* | | *VIT* |
| *ACTL6B* | | *CCDC17* | | *DPP4* | | *GRIK2* | *LRRD1* | | *OTOGL* | | | *RFX3* | | *SYNJ2* | | *VPS13A* |
| *ADAM23* | | *CCT3* | | *DRGX* | | *GUCY1A3* | *LRRFIP2* | | *OVCH1* | | | *RNF215* | | *TAF1B* | | *VPS33B* |
| *ADAM33* | | *CCT6B* | | *DUOX1* | | *GUCY2C* | *LRSAM1* | | *P4HB* | | | *RNF219* | | *TAF6* | | *VSIG4* |
| *ADAMTS12* | | *CD1E* | | *DYSF* | | *GYLTL1B* | *LTBP1* | | *PABPC4* | | | *RPL22* | | *TARBP1* | | *WAS* |
| *ADAMTS16* | | *CD300LF* | | *DZANK1* | | *HAAO* | *LUC7L2* | | *PACS2* | | | *RPL36A* | | *TBC1D1* | | *WASL* |
| *ADAMTS19* | | *CD5L* | | *ECHDC1* | | *HAP1* | *LUZP4* | | *PAEP* | | | *RPS5* | | *TBC1D21* | | *WDR44* |
| *ADAMTS20* | | *CD9* | | *EDN1* | | *HAUS5* | *MAEL* | | *PAGE1* | | | *RPS6KA1* | | *TBC1D3* | | *WDR52* |
| *ADAMTS5* | | *CD97* | | *EEF1A1* | | *HAUS6* | *MAGI1* | | *PARK2* | | | *RPTOR* | | *TBC1D5* | | *WDR62* |
| *ADAMTSL1* | | *CD99* | | *EFCAB5* | | *HCN1* | *MAN2A1* | | *PARP4* | | | *RPUSD4* | | *TBL1X* | | *WDR66* |
| *ADD2* | | *CDH18* | | *EFCAB6* | | *HDAC6* | *MAP2* | | *PCK2* | | | *RREB1* | | *TBP* | | *WDR72* |
| *AGMAT* | | *CDH24* | | *EFCAB7* | | *HEATR7B2* | *MAP2K4* | | *PCLO* | | | *RRP7A* | | *TBX15* | | *WDTC1* |
| *AGTPBP1* | | *CDH26* | | *EFHA2* | | *HECTD4* | *MAP3K1* | | *PCNT* | | | *RUNDC3A* | | *TBX22* | | *WLS* |
| *AHCTF1* | | *CDK11A* | | *EFNA5* | | *HECW1* | *MAP4K1* | | *PCNXL2* | | | *RUNX1* | | *TBX3* | | *WSCD2* |
| *AK5* | | *CDK12* | | *EIF1AX* | | *HECW2* | *MAPKAPK3* | | *PCSK5* | | | *RYR2* | | *TCF20* | | *WWP2* |
| *AKR1B10* | | *CDK14* | | *EIF2B5* | | *HID1* | *MAPRE3* | | *PCYT1A* | | | *RYR3* | | *TCF4* | | *XBP1* |
| *AKR1C1* | | *CDK18* | | *EIF2C2* | | *HIST1H3B* | *MAST1* | | *PDCD6* | | | *SAFB2* | | *TCP10* | | *XPO4* |
| *ALDH1A3* | | *CDK19* | | *EIF3E* | | *HLA-DRB1* | *MBIP* | | *PDE1C* | | | *SAG* | | *TCP11* | | *XPO5* |
| *ALDH2* | | *CDS1* | | *EIF3I* | | *HLA-DRB5* | *MBTPS2* | | *PDE2A* | | | *SAGE1* | | *TEK* | | *ZAP70* |
| *ALG5* | | *CEACAM20* | | *EIF4ENIF1* | | *HMCN1* | *MCF2L2* | | *PDE4DIP* | | | *SAMD8* | | *TERT* | | *ZBTB8OS* |
| *ALX4* | | *CECR2* | | *EIF4H* | | *HMHA1* | *MCOLN2* | | *PDIA5* | | | *SCN10A* | | *TESC* | | *ZC3H13* |
| *AMOT* | | *CELA2B* | | *ELAVL3* | | *HNF4A* | *MDGA2* | | *PDILT* | | | *SCN3A* | | *TEX35* | | *ZC3H7B* |
| *ANK2* | | *CGN* | | *ELL3* | | *HOMER2* | *MDN1* | | *PDRG1* | | | *SCN7A* | | *TFDP1* | | *ZDHHC11* |
| *ANKRD13D* | | *CHD3* | | *EMID2* | | *HPS3* | *MED23* | | *PEX6* | | | *SCN9A* | | *TGDS* | | *ZFC3H1* |
| *ANKRD20A4* | | *CHD4* | | *ENPP2* | | *HPS4* | *MEFV* | | *PGAP1* | | | *SDK2* | | *TGM2* | | *ZFR* |
| *ANKRD27* | | *CHD6* | | *ENTPD6* | | *HSPA12B* | *METTL14* | | *PHACTR3* | | | *SEC14L4* | | *TGM5* | | *ZMYM4* |
| *ANKRD28* | | *CHI3L1* | | *EPB41L2* | | *HSPD1* | *METTL5* | | *PHF20L1* | | | *SEC24B* | | *THBS2* | | *ZNF143* |
| *ANKRD30A* | | *CISD3* | | *EPB41L4B* | | *HYDIN* | *MGAM* | | *PHYH* | | | *SEH1L* | | *THEM5* | | *ZNF350* |
| *ANKRD30B* | | *CLCN7* | | *EPHB1* | | *IBSP* | *MICALL1* | | *PI4KB* | | | *SELP* | | *THOC1* | | *ZNF385A* |
| *ANKRD36B* | | *CLEC16A* | | *EPS8L3* | | *IFT172* | *MID1* | | *PIP4K2C* | | | *SEMA6A* | | *THSD7A* | | *ZNF414* |
| *ANO2* | | *CLINT1* | | *ESD* | | *IGSF9* | *MIER2* | | *PIP5K1C* | | | *SEPT12.* | | *THSD7B* | | *ZNF512B* |
| *AP1B1* | | *CNGB3* | | *ETNK2* | | *IKBKAP* | *MLL3* | | *PIWIL1* | | | *SERPINA7* | | *TIMD4* | | *ZNF541* |
| *AP1G2* | | *CNKSR2* | | *ETV6* | | *IKBKE* | *MLPH* | | *PKD1L2* | | | *SETD1B* | | *TIMM44* | | *ZNF563* |
| *AP3B1* | | *CNOT3* | | *EXOC4* | | *IL11RA* | *MORC1* | | *PKHD1* | | | *SETD2* | | *TIMP3* | | *ZNF614* |
| *APAF1* | | *CNOT4* | | *EXOC5* | | *IL13RA2* | *MORN1* | | *PKLR* | | | *SF1* | | *TJP3* | | *ZNF687* |
| *APLP2* | | *CNTN1* | | *EXOC6* | | *IL1RAPL1* | *MRPL1* | | *PLAC8* | | | *SF3B1* | | *TLE1* | | *ZNF705B* |
| *APMAP* | | *CNTN4* | | *EXOC7* | | *IL27RA* | *MRPL24* | | *PLCB4* | | | *SF3B14* | | *TLL1* | | *ZNF705G* |
| *APPL2* | | *CNTN5* | | *EXTL3* | | *IMPG1* | *MRPS18B* | | *PLCZ1* | | | *SF3B3* | | *TMC2* | | *ZNF711* |
| *AQP12A* | | *CNTNAP3B* | | *EYA4* | | *INHBA* | *MSI1* | | *PLEC* | | | *SGCZ* | | *TMED8* | | *ZNF804B* |
| *ARFGAP1* | | *CNTNAP5* | | *F8* | | *INPP5J* | *MTA2* | | *PLK2* | | | *SGIP1* | | *TMEM104* | | *ZSWIM8* |
| *ARFRP1* | | *COASY* | | *F9* | | *IQCA1* | *MTM1* | | *PLOD3* | | | *SGK1* | | *TMEM120B* | |  |
| *ARHGAP35* | | *COL14A1* | | *FAH* | | *ITFG2* | *MTR* | | *PLXNA1* | | | *SGPL1* | | *TMEM132D* | |  |
| *ARHGAP40* | | *COL16A1* | | *FAM114A2* | | *ITGA8* | *MTTP* | | *POLDIP2* | | | *SH2D3A* | | *TMEM145* | |  |
| *ARHGEF1* | | *COL19A1* | | *FAM131B* | | *ITGA9* | *MUC5B* | | *POLE* | | | *SH3BGR* | | *TMEM247* | |  |
| *ARHGEF7* | | *COL1A1* | | *FAM135B* | | *ITIH1* | *MUS81* | | *POLR2J* | | | *SH3PXD2A* | | *TMEM80* | |  |
| *ARNTL* | | *COL25A1* | | *FAM13C* | | *ITLN2* | *MYB* | | *POLR3B* | | | *SHISA4* | | *TMEM87A* | |  |
| *ARPC4-TTLL3* | | *COL4A5* | | *FAM157B* | | *ITM2A* | *MYBPC2* | | *POLR3GL* | | | *SI* | | *TMTC4* | |  |
| *ASH2L* | | *COL4A6* | | *FAM177B* | | *ITPKB* | *MYCBP2* | | *POLRMT* | | | *SIDT2* | | *TMX3* | |  |
| *ASTN1* | | *COL5A1* | | *FAM21A* | | *ITPR1* | *MYH15* | | *POM121L12* | | | *SIK3* | | *TNFAIP6* | |  |
| *ASXL2* | | *COL5A2* | | *FAM3A* | | *KCNAB2* | *MYH2* | | *POTEG* | | | *SIM1* | | *TNFSF4* | |  |
| *ATAD2B* | | *COL5A3* | | *FAM49A* | | *KCNH6* | *MYH4* | | *PPA1* | | | *SIM2* | | *TNN* | |  |
| *ATG9B* | | *COL6A5* | | *FAM49B* | | *KCNQ2* | *MYH8* | | *PPDPF* | | | *SLC13A3* | | *TNNT1* | |  |
| *ATP10B* | | *COL6A6* | | *FAM5C* | | *KDM4A* | *MYH9* | | *PPEF1* | | | *SLC17A6* | | *TNR* | |  |
| *ATP10D* | | *COL9A1* | | *FAM86B1* | | *KDM6A* | *MYL5* | | *PPFIBP2* | | | *SLC17A8* | | *TNS3* | |  |
| *ATP12A* | | *COPA* | | *FAN1* | | *KEAP1* | *MYL6* | | *PPIL2* | | | *SLC25A1* | | *TP53BP1* | |  |
| *ATP2C1* | | *COPG1* | | *FANCC* | | *KIAA0195* | *MYLK2* | | *PPP1R17* | | | *SLC25A30* | | *TPCN1* | |  |
| *ATP6V0A2* | | *CPA1* | | *FASTK* | | *KIAA0226* | *MYO3A* | | *PPP4R4* | | | *SLC26A3* | | *TPH2* | |  |
| *ATP8B2* | | *CPSF3* | | *FATE1* | | *KIAA0319* | *MYOM1* | | *PQBP1* | | | *SLC2A2* | | *TPMT* | |  |
| *ATXN2* | | *CPSF6* | | *FBN2* | | *KIAA0922* | *NACAD* | | *PREB* | | | *SLC30A5* | | *TPTE* | |  |
| *ATXN7L2* | | *CRTAM* | | *FDCSP* | | *KIAA1191* | *NARF* | | *PREX2* | | | *SLC35B2* | | *TRIM33* | |  |
| *BAX* | | *CRTAP* | | *FLNC* | | *KIAA1199* | *NAT10* | | *PRKACA* | | | *SLC35B4* | | *TRIM51* | |  |
| *BBS9* | | *CRYBG3* | | *FLOT2* | | *KIAA1211L* | *NAV3* | | *PRKAG3* | | | *SLC38A4* | | *TRIM58* | |  |
| *BCAS1* | | *CSMD1* | | *FLT3LG* | | *KIF13A* | *NBPF1* | | *PRKCD* | | | *SLC38A5* | | *TRIML1* | |  |
| *BCAS2* | | *CSMD3* | | *FMN2* | | *KIF1B* | *NBPF10* | | *PRKDC* | | | *SLC43A1* | | *TRIO* | |  |
| *BCL2L11* | | *CSN3* | | *FMNL3* | | *KIF26B* | *NCF2* | | *PRKX* | | | *SLC45A1* | | *TRIP11* | |  |
| *BCR* | | *CSNK1E* | | *FNDC4* | | *KIF5B* | *NCKAP1* | | *PRRX1* | | | *SLC4A10* | | *TRMT112* | |  |
| *BLOC1S1* | | *CSPP1* | | *FNIP2* | | *KIFAP3* | *NCOR1* | | *PRSS1* | | | *SLC4A4* | | *TRPC5* | |  |
| *BMPR1B* | | *CTCF* | | *FOLH1* | | *KIFC1* | *NCOR2* | | *PRUNE* | | | *SLC5A1* | | *TRUB1* | |  |
| *BRF1* | | *CTIF* | | *FOXJ2* | | *KIR2DL3* | *NEK5* | | *PSG2* | | | *SLC6A5* | | *TSGA10* | |  |
| *BRSK2* | | *CTNNA2* | | *FRG1* | | *KIR3DL3* | *NELL1* | | *PSG5* | | | *SLC8A1* | | *TSKS* | |  |
| *BRWD3* | | *CTSF* | | *FRG2B* | | *KLHL1* | *NFE2L2* | | *PSIP1* | | | *SLCO1B7* | | *TSPAN12* | |  |
| *BSG* | | *CYP2A13* | | *FRMD4A* | | *KLHL14* | *NIPBL* | | *PSMC4* | | | *SLCO5A1* | | *TSR2* | |  |
| *BTNL3* | | *CYP3A4* | | *FRMPD2* | | *KLK1* | *NLGN3* | | *PSMC6* | | | *SMTN* | | *TTF2* | |  |
| *BTRC* | | *CYP4A11* | | *FRMPD4* | | *KMT2B* | *NLRC3* | | *PSTPIP1* | | | *SNTG1* | | *TTN* | |  |
| *C12orf5* | | *CYTH4* | | *FSD2* | | *KMT2C* | *NLRP4* | | *PTBP3* | | | *SORCS3* | | *TUBA3C* | |  |
| *C19orf38* | | *DCLK2* | | *FSHR* | | *KRT2* | *NMI* | | *PTCD3* | | | *SPAG16* | | *TUBGCP4* | |  |
| *C1orf112* | | *DCST1* | | *FUBP1* | | *KRT9* | *NOP2* | | *PTGES3L-AARSD1* | | | *SPATA13* | | *TUBGCP5* | |  |
| *C1orf35* | | *DDB1* | | *FUNDC1* | | *KRTAP5-5* | *NOS1* | | *PTGS2* | | | *SPG20* | | *TYK2* | |  |
| *C20orf112* | | *DDX24* | | *GAB3* | | *KTN1* | *NOS2* | | *PTPLAD1* | | | *SPINT1* | | *TYRP1* | |  |
| *C2orf47* | | *DDX3X* | | *GABRD* | | *L3MBTL1* | *NRXN1* | | *PTPN13* | | | *SPPL2A* | | *U2AF1* | |  |
| *C2orf62* | | *DEPDC4* | | *GAD2* | | *LARP1* | *NRXN2* | | *PTPRA* | | | *SPPL3* | | *U2AF2* | |  |
| *C7orf53* | | *DGKK* | | *GALNT13* | | *LCN10* | *NT5C3L* | | *PTPRD* | | | *SPRED1* | | *UBASH3A* | |  |
| *C9orf114* | | *DHCR24* | | *GALNT14* | | *LCT* | *NTM* | | *PTPRM* | | | *SPTA1* | | *UBE2Q1* | |  |
| *C9orf43* | | *DHDDS* | | *GFRAL* | | *LCTL* | *NUDCD2* | | *PYHIN1* | | | *SRRT* | | *UBE4B* | |  |
| *CACNA1A* | | *DHX9* | | *GIGYF1* | | *LETM1* | *NUP205* | | *QRICH2* | | | *SSBP3* | | *UCHL3* | |  |
| *CACNA1D* | | *DIAPH1* | | *GINS4* | | *LGALS13* | *NUP210* | | *RAB1B* | | | *SSH2* | | *UCK2* | |  |
| *CACNA1E* | | *DKC1* | | *GIPR* | | *LILRB3* | *NUTM1* | | *RAB3GAP2* | | | *SSPO* | | *UGT8* | |  |
| *CADM2* | | *DLST* | | *GKN2* | | *LILRB4* | *NWD1* | | *RAB6A* | | | *ST18* | | *ULK3* | |  |
| *CAMKK1* | | *DMD* | | *GLB1L3* | | *LIPN* | *NXF1* | | *RAC2* | | | *ST6GALNAC1* | | *UMOD* | |  |

Supplementary table S2. Sequencing Sample Quality Control Information.

| Quality control item Sample type | Tumor | Normal Control |
| --- | --- | --- |
|  | FFPE tissue | FFPE tissue |
| Tumor component content | >=10% | - |
| Base quality >=Q30 percentage | >=80% | >=80% |
| Average Sequencing Depth (with dup) | >=500 | >=500 |
| Average Sequencing Depth (without dup) | >=500 | >=500 |
| Proportion of bases above 0.2x average depth | >=90% | >=90% |
| Genome matching rate | >=90% | >=90% |
| Sample pairwise agreement rate | >=90% | >=90% |
